# Supplementary material for: Estimating the effect of a scanner upgrade on measures of grey matter structure for longitudinal designs
Source: PLoS One. 2021 Oct 5;16(10):e0239021. doi: 10.1371/journal.pone.0239021 (PMC8491918; doi:10.1371/journal.pone.0239021)
Supplement: S1 File — (PDF) [file pone.0239021.s001.pdf]

Supplementary Material for 'Estimating the effect of a scanner upgrade on measures of grey matter structure for longitudinal designs'

Evelyn Medawar, MSc [1][2][3] Ronja Thieleking, MSc [1]
Iryna Manuilova, BSc [1] Maria Paerisch[1] Arno Villringer, Prof. [1][2][3][4][5]
A. Veronica Witte, PhD [1][4][5] Frauke Beyer, PhD [1][4]

1 Max-Planck-Institute for Human Cognitive and Brain Sciences, Leipzig
2 Berlin School of Mind and Brain, Humboldt-Universität zu Berlin, Berlin, Germany
3 Center for Stroke Research Berlin (CSB), Charité Universitätsmedizin, Berlin, Germany
4 CRC 1052 "Obesity Mechanisms," Subproject A1, Leizpig University, Leipzig, Germany
5 Day Clinic for Cognitive Neurology, University of Leipzig Medical Center - Leipzig University, Leipzig, Germany

Contents

1 Bland-Altman plot for all subcortical ROI **2**

2 Cortical thickness **3**
2.1 Vertex-wise analysis of cortical thickness ICC . . . 3
2.2 Vertex-wise analysis of cortical thickness PD . . . 3
2.3 Reliability and percent difference table of cortical thickness . . . 3

3 Cortical area **5**
3.1 Vertex-wise analysis of cortical area ICC . . . 5
3.2 Vertex-wise analysis of cortical area PD . . . 5
3.3 Reliability and percent difference table of cortical area . . . 5

4 Cortical volume **7**
4.1 Vertex-wise analysis of cortical volume ICC . . . 7
4.2 Vertex-wise analysis of cortical volume PD . . . 7
4.3 Reliability and percent difference table of cortical volume . . . 7

5 Correlation of CNR and CT **9**

6 Gradunwarp gradient distortion corrected data **11**
6.1 Vertex-wise analysis of gradunwarp distortion-corrected ICC . . . 11
6.2 Vertex-wise analysis of gradunwarp distortion-corrected PD . . . 11

7 Skyra D versus Verio ND analysis **11**

8 QA differences **11**

1 Bland-Altman plot for all subcortical ROI

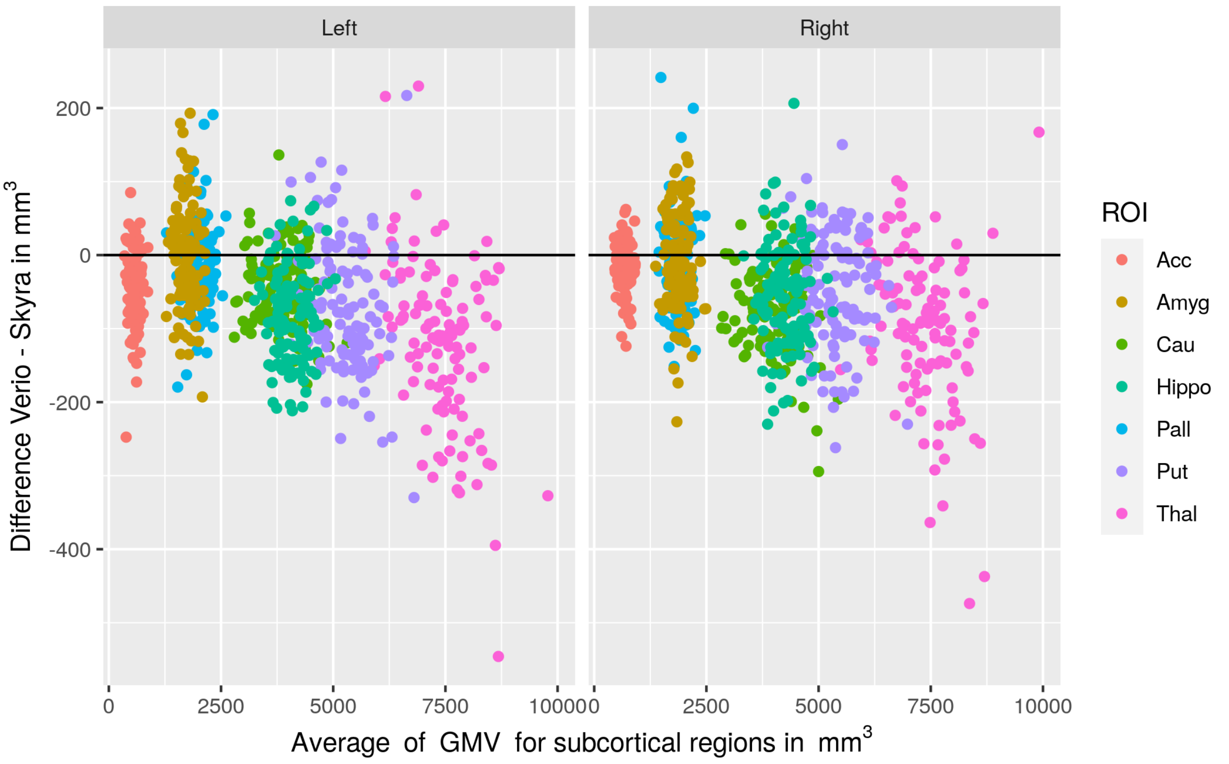

Figure 1: Bland-Altman plot showing differences of Verio-Skyra against means for all subcortical ROI. Left/right panel show left/right hemisphere.)

2 Cortical thickness

2.1 Vertex-wise analysis of cortical thickness ICC

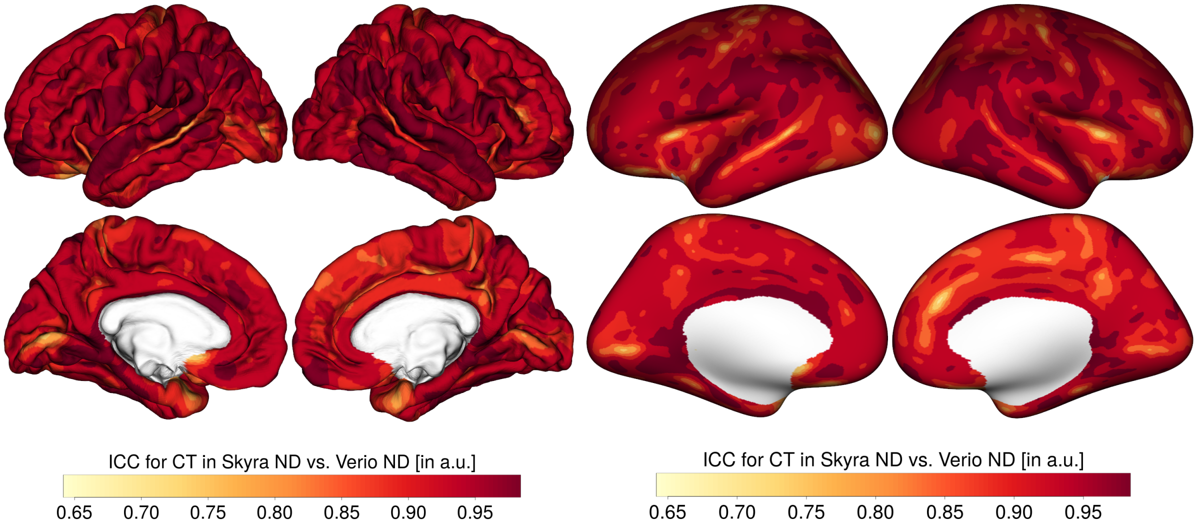

Figure 2: ICC for cortical thickness (CT) on white surface (left panel) and inflated surface (right panel)

2.2 Vertex-wise analysis of cortical thickness PD

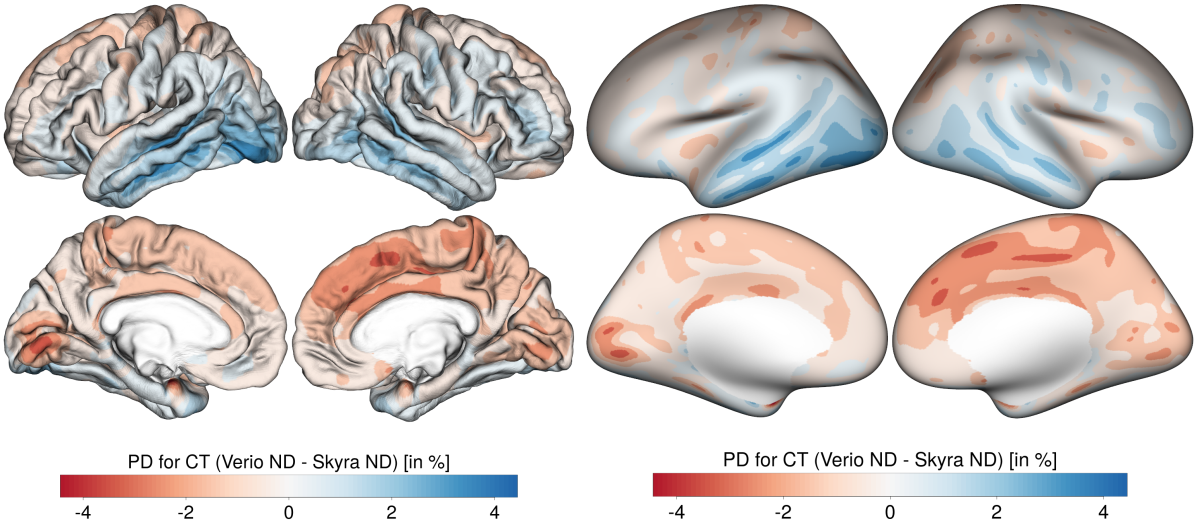

Figure 3: PD for cortical thickness (CT) on white surface (left panel) and inflated surface (right panel). Positive values: Verio>Skyra , negative values: Verio<Skyra))

2.3 Reliability and percent difference table of cortical thickness

Table 1: Reliability and differences for cortical thickness

ROI	hemi	ICC	lower ICC	upper ICC	PD	T	p	adj.p
bankssts	lh	0.834	0.777	0.839	1.71	7.55	0.00	**0**
bankssts	rh	0.928	0.914	0.939	1.32	8.83	0.00	**0**
caudalanteriorcingulate	lh	0.967	0.964	0.973	-0.94	-5.64	0.00	**0**
caudalanteriorcingulate	rh	0.912	0.903	0.930	-2.01	-10.62	0.00	**0**
caudalmiddlefrontal	lh	0.921	0.904	0.935	-0.38	-2.49	0.01	**0.02**
caudalmiddlefrontal	rh	0.906	0.876	0.926	-0.16	-0.94	0.35	0.44
cuneus	lh	0.906	0.858	0.933	-0.87	-3.92	0.00	**0**
cuneus	rh	0.931	0.917	0.930	-1.15	-6.20	0.00	**0**
entorhinal	lh	0.923	0.901	0.938	0.18	0.68	0.50	0.58
entorhinal	rh	0.942	0.925	0.948	-0.31	-1.18	0.24	0.31
fusiform	lh	0.888	0.867	0.893	0.52	3.10	0.00	**0**
fusiform	rh	0.919	0.861	0.945	0.25	1.87	0.06	0.09
inferiorparietal	lh	0.881	0.851	0.913	0.34	2.09	0.04	0.06
inferiorparietal	rh	0.931	0.928	0.940	-0.36	-2.71	0.01	**0.02**
inferiortemporal	lh	0.865	0.825	0.874	1.45	8.51	0.00	**0**
inferiortemporal	rh	0.915	0.877	0.946	1.06	8.56	0.00	**0**
isthmuscingulate	lh	0.971	0.969	0.978	-0.89	-5.49	0.00	**0**
isthmuscingulate	rh	0.937	0.913	0.948	-1.05	-5.69	0.00	**0**
lateraloccipital	lh	0.828	0.781	0.833	1.79	8.62	0.00	**0**
lateraloccipital	rh	0.918	0.880	0.940	0.61	3.46	0.00	**0**
lateralorbitofrontal	lh	0.859	0.825	0.890	0.48	2.56	0.01	**0.02**
lateralorbitofrontal	rh	0.816	0.777	0.834	0.39	1.53	0.13	0.18
lingual	lh	0.935	0.916	0.930	-0.90	-5.32	0.00	**0**
lingual	rh	0.906	0.876	0.925	-1.02	-5.30	0.00	**0**
medialorbitofrontal	lh	0.858	0.842	0.891	0.51	1.84	0.07	0.11
medialorbitofrontal	rh	0.888	0.866	0.907	-0.08	-0.35	0.73	0.77
middletemporal	lh	0.883	0.859	0.913	1.55	9.83	0.00	**0**
middletemporal	rh	0.921	0.903	0.924	1.16	9.44	0.00	**0**
parahippocampal	lh	0.974	0.970	0.977	0.48	2.93	0.00	**0**
parahippocampal	rh	0.961	0.952	0.964	-0.05	-0.33	0.74	0.77
paracentral	lh	0.892	0.839	0.886	-1.43	-8.33	0.00	**0**
paracentral	rh	0.826	0.816	0.866	-2.06	-10.80	0.00	**0**
parsopercularis	lh	0.936	0.924	0.947	-0.22	-1.65	0.10	0.14
parsopercularis	rh	0.947	0.938	0.964	0.11	0.73	0.47	0.55
parsorbitalis	lh	0.911	0.883	0.942	0.00	0.08	0.94	0.95
parsorbitalis	rh	0.929	0.910	0.946	0.09	0.43	0.67	0.73
parstriangularis	lh	0.894	0.840	0.902	-0.14	-0.83	0.41	0.51
parstriangularis	rh	0.908	0.874	0.916	0.08	0.43	0.66	0.73
pericalcarine	lh	0.862	0.786	0.897	-1.44	-4.58	0.00	**0**
pericalcarine	rh	0.882	0.825	0.913	-1.42	-4.27	0.00	**0**
postcentral	lh	0.950	0.938	0.963	-0.38	-2.76	0.01	**0.02**
postcentral	rh	0.940	0.930	0.961	-0.23	-1.36	0.18	0.24
posteriorcingulate	lh	0.938	0.933	0.954	-1.21	-7.89	0.00	**0**
posteriorcingulate	rh	0.879	0.847	0.884	-1.94	-12.90	0.00	**0**
precentral	lh	0.918	0.900	0.932	-0.56	-3.98	0.00	**0**
precentral	rh	0.960	0.954	0.969	-0.18	-1.31	0.19	0.25
precuneus	lh	0.863	0.833	0.891	-0.89	-5.13	0.00	**0**
precuneus	rh	0.845	0.823	0.867	-1.42	-9.32	0.00	**0**
rostralanteriorcingulate	lh	0.899	0.855	0.922	-0.33	-1.52	0.13	0.18
rostralanteriorcingulate	rh	0.919	0.870	0.945	-0.92	-5.02	0.00	**0**
rostralmiddlefrontal	lh	0.886	0.874	0.912	-0.44	-2.78	0.01	**0.02**
rostralmiddlefrontal	rh	0.873	0.811	0.898	-0.07	-0.40	0.69	0.74
superiorfrontal	lh	0.891	0.892	0.922	-1.00	-7.10	0.00	**0**
superiorfrontal	rh	0.816	0.812	0.855	-1.59	-10.79	0.00	**0**
superiorparietal	lh	0.917	0.918	0.939	-0.54	-3.62	0.00	**0**

3 Cortical area

3.1 Vertex-wise analysis of cortical area ICC

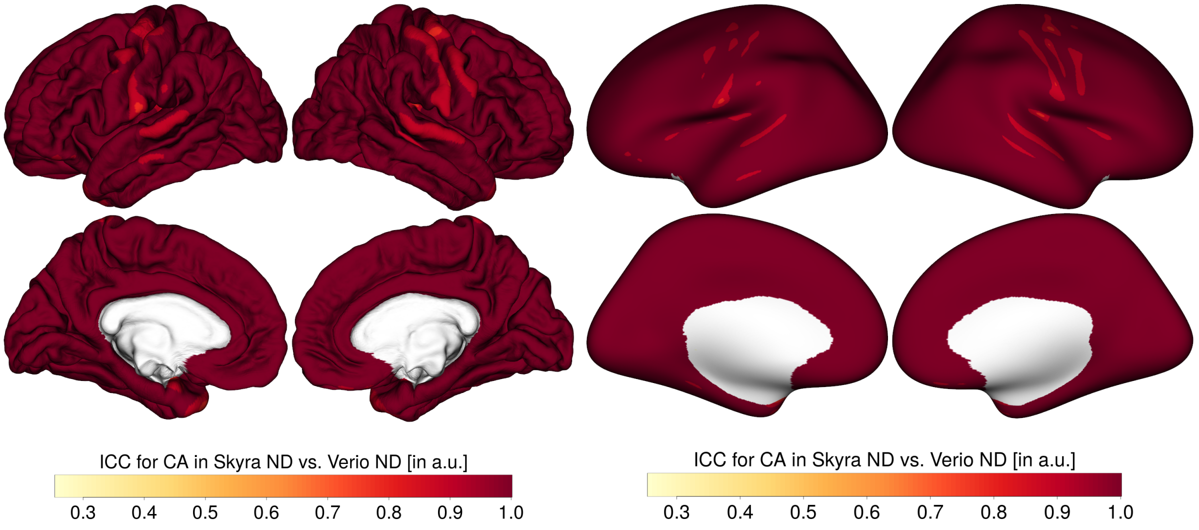

Figure 4: ICC for cortical area (CA) on white surface (left panel) and inflated surface (right panel)

3.2 Vertex-wise analysis of cortical area PD

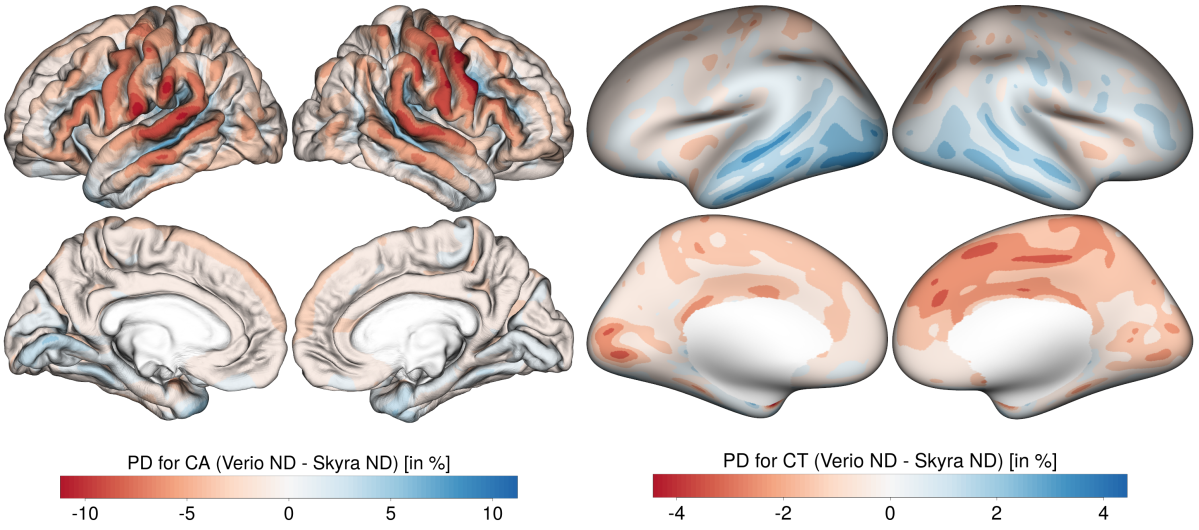

Figure 5: PD for cortical area (CA) on white surface (left panel) and inflated surface (right panel). Positive values: Verio>Skyra , negative values: Verio<Skyra))

3.3 Reliability and percent difference table of cortical area

Table 2: Reliability and differences for cortical area

ROI	hemi	ICC	lower ICC	upper ICC	PD	T	p	adj.p
bankssts	lh	0.992	0.991	0.993	1.72	12.35	0.00	**0**
bankssts	rh	0.988	0.989	0.989	1.95	16.67	0.00	**0**
caudalanteriorcingulate	lh	0.994	0.993	0.995	-0.61	-3.30	0.00	**0**
caudalanteriorcingulate	rh	0.993	0.993	0.995	-1.02	-5.42	0.00	**0**
caudalmiddlefrontal	lh	0.997	0.995	0.997	-0.61	-6.10	0.00	**0**
caudalmiddlefrontal	rh	0.997	0.996	0.998	-0.47	-4.48	0.00	**0**
cuneus	lh	0.992	0.991	0.993	0.19	0.90	0.37	0.4
cuneus	rh	0.993	0.994	0.995	-0.32	-2.12	0.04	0.05
entorhinal	lh	0.980	0.979	0.988	-1.61	-4.15	0.00	**0**
entorhinal	rh	0.980	0.977	0.985	0.31	0.78	0.43	0.45
fusiform	lh	0.995	0.994	0.996	-0.68	-6.65	0.00	**0**
fusiform	rh	0.997	0.996	0.998	-0.24	-2.81	0.01	**0.01**
inferiorparietal	lh	0.996	0.995	0.997	-0.75	-7.64	0.00	**0**
inferiorparietal	rh	0.995	0.993	0.995	-0.99	-10.65	0.00	**0**
inferiortemporal	lh	0.995	0.995	0.997	-0.79	-7.02	0.00	**0**
inferiortemporal	rh	0.996	0.995	0.997	-0.45	-4.38	0.00	**0**
isthmuscingulate	lh	0.993	0.991	0.996	-0.26	-1.21	0.23	0.25
isthmuscingulate	rh	0.986	0.984	0.989	-0.80	-3.85	0.00	**0**
lateraloccipital	lh	0.993	0.990	0.994	-1.21	-13.98	0.00	**0**
lateraloccipital	rh	0.994	0.995	0.995	-0.90	-9.34	0.00	**0**
lateralorbitofrontal	lh	0.992	0.991	0.995	0.10	0.87	0.39	0.41
lateralorbitofrontal	rh	0.955	0.939	0.961	-0.99	-3.07	0.00	**0**
lingual	lh	0.991	0.989	0.992	0.85	5.66	0.00	**0**
lingual	rh	0.992	0.988	0.994	0.05	0.29	0.77	0.77
medialorbitofrontal	lh	0.945	0.928	0.961	-0.83	-2.24	0.03	**0.04**
medialorbitofrontal	rh	0.944	0.936	0.961	-1.68	-5.67	0.00	**0**
middletemporal	lh	0.984	0.981	0.988	-2.38	-24.65	0.00	**0**
middletemporal	rh	0.987	0.984	0.987	-1.77	-22.71	0.00	**0**
parahippocampal	lh	0.985	0.985	0.989	-0.10	-0.63	0.53	0.54
parahippocampal	rh	0.987	0.986	0.990	-0.81	-5.24	0.00	**0**
paracentral	lh	0.988	0.986	0.990	-1.21	-8.87	0.00	**0**
paracentral	rh	0.985	0.983	0.986	-1.28	-8.43	0.00	**0**
parsopercularis	lh	0.995	0.993	0.997	-0.86	-8.54	0.00	**0**
parsopercularis	rh	0.995	0.993	0.996	-0.54	-4.63	0.00	**0**
parsorbitalis	lh	0.952	0.945	0.958	-3.42	-19.97	0.00	**0**
parsorbitalis	rh	0.975	0.963	0.976	-2.39	-13.74	0.00	**0**
parstriangularis	lh	0.991	0.989	0.992	-1.70	-15.83	0.00	**0**
parstriangularis	rh	0.988	0.985	0.988	-2.14	-16.09	0.00	**0**
pericalcarine	lh	0.996	0.994	0.996	-0.72	-5.43	0.00	**0**
pericalcarine	rh	0.993	0.987	0.993	-1.10	-7.38	0.00	**0**
postcentral	lh	0.971	0.968	0.973	-2.29	-16.89	0.00	**0**
postcentral	rh	0.973	0.966	0.981	-2.29	-15.76	0.00	**0**
posteriorcingulate	lh	0.994	0.992	0.995	-0.80	-6.16	0.00	**0**
posteriorcingulate	rh	0.992	0.987	0.993	-0.97	-7.22	0.00	**0**
precentral	lh	0.983	0.981	0.986	-1.51	-12.23	0.00	**0**
precentral	rh	0.984	0.978	0.988	-1.32	-10.89	0.00	**0**
precuneus	lh	0.992	0.992	0.994	-1.22	-12.88	0.00	**0**
precuneus	rh	0.992	0.989	0.993	-1.42	-14.01	0.00	**0**
rostralanteriorcingulate	lh	0.979	0.972	0.983	-1.84	-5.55	0.00	**0**
rostralanteriorcingulate	rh	0.990	0.985	0.992	-1.60	-7.79	0.00	**0**
rostralmiddlefrontal	lh	0.994	0.992	0.996	-0.97	-9.70	0.00	**0**
rostralmiddlefrontal	rh	0.993	0.991	0.994	-0.91	-6.78	0.00	**0**
superiorfrontal	lh	0.987	0.984	0.988	-1.41	-12.36	0.00	**0**
superiorfrontal	rh	0.989	0.987	0.989	-1.36	-11.55	0.00	**0**
superiorparietal	lh	0.993	0.990	0.994	-1.04	-8.93	0.00	**0**

4 Cortical volume

4.1 Vertex-wise analysis of cortical volume ICC

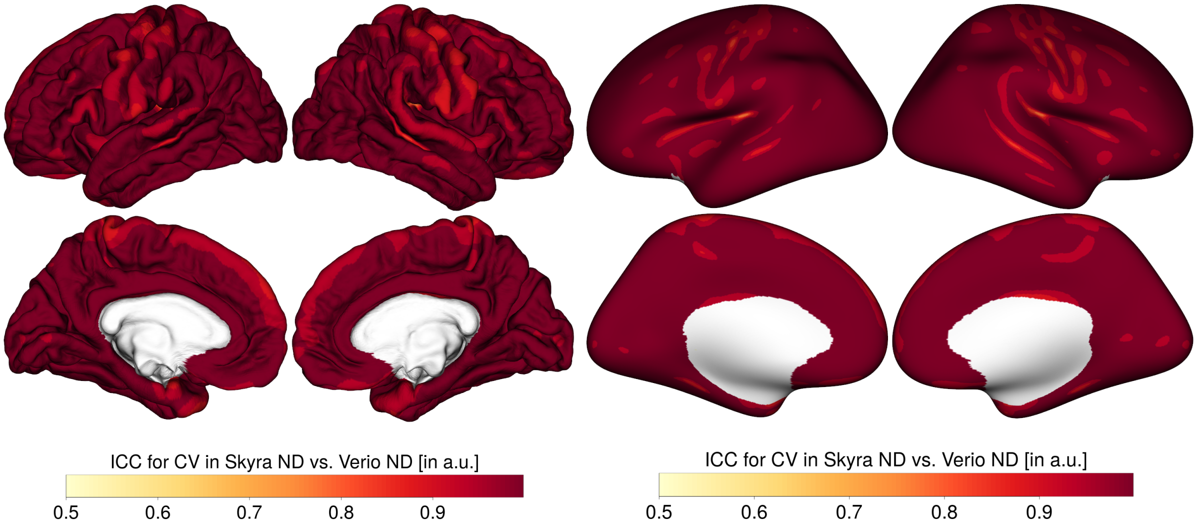

Figure 6: ICC for cortical volume (CV) on white surface (left panel) and inflated surface (right panel)

4.2 Vertex-wise analysis of cortical volume PD

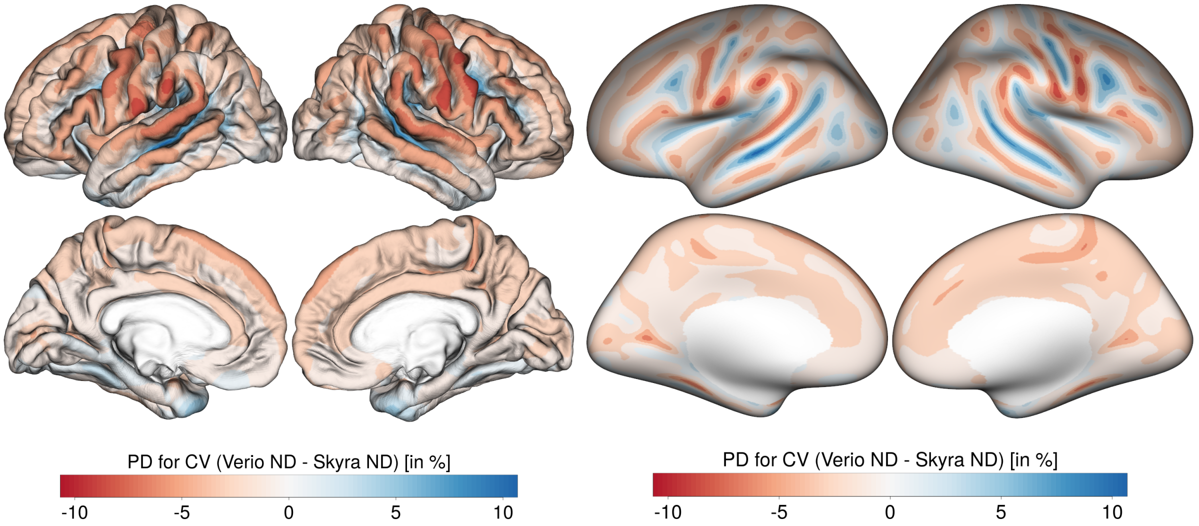

Figure 7: PD for cortical volume (CV) on white surface (left panel) and inflated surface (right panel). Positive values: Verio>Skyra , negative values: Verio<Skyra))

4.3 Reliability and percent difference table of cortical volume

Table 3: Reliability and differences for cortical volume

ROI	hemi	ICC	lower ICC	upper ICC	PD	T	p	adj.p
bankssts	lh	0.984	0.978	0.988	1.98	6.80	0.00	**0**
bankssts	rh	0.987	0.983	0.989	2.20	12.44	0.00	**0**
caudalanteriorcingulate	lh	0.992	0.991	0.994	-2.08	-9.17	0.00	**0**
caudalanteriorcingulate	rh	0.986	0.981	0.988	-3.05	-14.55	0.00	**0**
caudalmiddlefrontal	lh	0.983	0.976	0.983	-1.75	-8.06	0.00	**0**
caudalmiddlefrontal	rh	0.983	0.981	0.985	-1.62	-6.61	0.00	**0**
cuneus	lh	0.987	0.981	0.989	-1.02	-4.29	0.00	**0**
cuneus	rh	0.988	0.984	0.989	-1.48	-7.37	0.00	**0**
entorhinal	lh	0.956	0.944	0.966	-1.23	-2.75	0.01	**0.01**
entorhinal	rh	0.960	0.933	0.965	0.19	0.33	0.74	0.74
fusiform	lh	0.986	0.982	0.988	-0.36	-1.43	0.16	0.18
fusiform	rh	0.992	0.989	0.991	0.09	0.69	0.49	0.51
inferiorparietal	lh	0.986	0.979	0.991	-1.29	-6.06	0.00	**0**
inferiorparietal	rh	0.982	0.981	0.984	-2.35	-14.79	0.00	**0**
inferiortemporal	lh	0.988	0.986	0.991	-0.97	-4.05	0.00	**0**
inferiortemporal	rh	0.993	0.989	0.993	-0.36	-1.88	0.06	0.07
isthmuscingulate	lh	0.993	0.990	0.994	-0.78	-4.07	0.00	**0**
isthmuscingulate	rh	0.986	0.983	0.988	-1.50	-6.92	0.00	**0**
lateraloccipital	lh	0.981	0.972	0.985	-0.27	-0.78	0.44	0.47
lateraloccipital	rh	0.986	0.978	0.989	-1.07	-4.79	0.00	**0**
lateralorbitofrontal	lh	0.984	0.984	0.989	-0.26	-1.08	0.28	0.3
lateralorbitofrontal	rh	0.971	0.961	0.979	-1.06	-3.87	0.00	**0**
lingual	lh	0.990	0.988	0.993	-0.74	-3.97	0.00	**0**
lingual	rh	0.986	0.984	0.989	-1.35	-6.78	0.00	**0**
medialorbitofrontal	lh	0.975	0.968	0.978	-0.43	-1.54	0.13	0.15
medialorbitofrontal	rh	0.964	0.951	0.971	-1.61	-6.12	0.00	**0**
middletemporal	lh	0.981	0.970	0.983	-2.20	-10.56	0.00	**0**
middletemporal	rh	0.983	0.975	0.988	-1.95	-12.98	0.00	**0**
parahippocampal	lh	0.986	0.982	0.985	0.08	0.39	0.70	0.71
parahippocampal	rh	0.983	0.979	0.986	-0.64	-2.90	0.00	**0**
paracentral	lh	0.962	0.961	0.970	-2.57	-10.25	0.00	**0**
paracentral	rh	0.952	0.949	0.959	-2.71	-10.11	0.00	**0**
parsopercularis	lh	0.985	0.979	0.987	-1.73	-9.05	0.00	**0**
parsopercularis	rh	0.990	0.989	0.994	-1.13	-5.80	0.00	**0**
parsorbitalis	lh	0.958	0.945	0.968	-3.30	-13.55	0.00	**0**
parsorbitalis	rh	0.967	0.958	0.968	-2.72	-10.78	0.00	**0**
parstriangularis	lh	0.980	0.977	0.982	-2.33	-10.92	0.00	**0**
parstriangularis	rh	0.981	0.977	0.983	-2.65	-12.87	0.00	**0**
pericalcarine	lh	0.965	0.943	0.968	-3.20	-8.99	0.00	**0**
pericalcarine	rh	0.962	0.959	0.977	-2.92	-7.97	0.00	**0**
postcentral	lh	0.956	0.931	0.959	-3.08	-13.11	0.00	**0**
postcentral	rh	0.953	0.948	0.958	-2.96	-10.56	0.00	**0**
posteriorcingulate	lh	0.986	0.985	0.989	-1.97	-11.24	0.00	**0**
posteriorcingulate	rh	0.975	0.972	0.978	-2.53	-13.53	0.00	**0**
precentral	lh	0.957	0.951	0.965	-2.72	-12.50	0.00	**0**
precentral	rh	0.961	0.950	0.968	-2.46	-10.94	0.00	**0**
precuneus	lh	0.975	0.969	0.973	-2.06	-9.51	0.00	**0**
precuneus	rh	0.971	0.969	0.977	-2.57	-12.81	0.00	**0**
rostralanteriorcingulate	lh	0.986	0.983	0.988	-2.03	-7.24	0.00	**0**
rostralanteriorcingulate	rh	0.986	0.980	0.985	-2.43	-10.62	0.00	**0**
rostralmiddlefrontal	lh	0.976	0.972	0.979	-2.35	-12.33	0.00	**0**
rostralmiddlefrontal	rh	0.976	0.966	0.974	-2.33	-10.82	0.00	**0**
superiorfrontal	lh	0.950	0.940	0.954	-2.87	-12.51	0.00	**0**
superiorfrontal	rh	0.951	0.924	0.965	-3.07	-13.03	0.00	**0**
superiorparietal	lh	0.973	0.961	0.974	-2.17	-8.91	0.00	**0**

5 Correlation of CNR and CT

Table 4: Association of CNR and scanner with CT

ROI	hemi	CNR estimate	scanner estimate	p CNR	p scanner	adj.p.CNR	adj.p.scanner
bankssts	lh	0.092	0.036	0.000	0.00	**0**	**0**
bankssts	rh	0.081	0.026	0.000	0.00	**0**	**0**
caudalanteriorcingulate	lh	0.030	-0.028	0.000	0.00	0.159	0.18
caudalanteriorcingulate	rh	-0.016	-0.051	0.000	0.00	0.541	0.57
caudalmiddlefrontal	lh	0.034	-0.013	0.003	0.01	**0.041**	0.06
caudalmiddlefrontal	rh	0.060	-0.011	0.032	0.05	**0.005**	**0.01**
cuneus	lh	0.058	-0.023	0.000	0.00	**0.002**	**0.01**
cuneus	rh	0.044	-0.026	0.000	0.00	**0.032**	0.05
entorhinal	lh	0.081	-0.001	0.920	0.92	**0.033**	0.05
entorhinal	rh	0.083	-0.017	0.137	0.17	0.088	0.11
fusiform	lh	0.073	0.008	0.090	0.13	**0**	**0**
fusiform	rh	0.052	0.001	0.815	0.84	**0.006**	**0.01**
inferiorparietal	lh	0.076	0.002	0.668	0.70	**0**	**0**
inferiorparietal	rh	0.046	-0.016	0.000	0.00	**0.009**	**0.02**
inferiortemporal	lh	0.074	0.035	0.000	0.00	**0**	**0**
inferiortemporal	rh	0.039	0.026	0.000	0.00	**0.027**	0.05
isthmuscingulate	lh	0.036	-0.025	0.000	0.00	**0.049**	0.07
isthmuscingulate	rh	0.089	-0.034	0.000	0.00	**0**	**0**
lateraloccipital	lh	0.085	0.034	0.000	0.00	**0**	**0**
lateraloccipital	rh	0.061	0.008	0.148	0.18	**0.004**	**0.01**
lateralorbitofrontal	lh	0.059	0.007	0.153	0.18	**0.001**	**0**
lateralorbitofrontal	rh	0.055	0.002	0.835	0.85	0.058	0.08
lingual	lh	0.033	-0.023	0.000	0.00	**0.041**	0.06
lingual	rh	0.031	-0.025	0.000	0.00	0.152	0.17
medialorbitofrontal	lh	0.012	0.011	0.117	0.15	0.62	0.63
medialorbitofrontal	rh	0.045	-0.008	0.229	0.27	0.073	0.09
middletemporal	lh	0.071	0.039	0.000	0.00	**0**	**0**
middletemporal	rh	0.052	0.028	0.000	0.00	**0.008**	**0.02**
parahippocampal	lh	0.044	0.010	0.051	0.08	0.052	0.07
parahippocampal	rh	0.076	-0.012	0.057	0.09	**0.007**	**0.02**
paracentral	lh	0.037	-0.040	0.000	0.00	**0.047**	0.07
paracentral	rh	-0.003	-0.055	0.000	0.00	0.899	0.9
parsopercularis	lh	0.032	-0.009	0.026	0.05	**0.041**	0.06
parsopercularis	rh	0.037	-0.003	0.489	0.53	0.05	0.07
parsorbitalis	lh	0.065	-0.005	0.382	0.43	**0.005**	**0.01**
parsorbitalis	rh	0.093	-0.010	0.113	0.15	**0.001**	**0**
parstriangularis	lh	0.045	-0.008	0.112	0.15	**0.011**	**0.02**
parstriangularis	rh	0.071	-0.008	0.105	0.15	**0**	**0**
pericalcarine	lh	0.051	-0.032	0.000	0.00	**0.026**	0.05
pericalcarine	rh	0.048	-0.026	0.001	0.00	0.115	0.14
postcentral	lh	0.051	-0.013	0.000	0.00	**0**	**0**
postcentral	rh	0.042	-0.009	0.049	0.08	**0.021**	**0.04**
posteriorcingulate	lh	0.022	-0.032	0.000	0.00	0.202	0.23
posteriorcingulate	rh	-0.012	-0.049	0.000	0.00	0.501	0.53
precentral	lh	0.037	-0.019	0.000	0.00	**0.024**	**0.04**
precentral	rh	0.039	-0.010	0.019	0.03	**0.032**	0.05
precuneus	lh	0.058	-0.027	0.000	0.00	**0.001**	**0**
precuneus	rh	0.041	-0.041	0.000	0.00	**0.031**	0.05
rostralanteriorcingulate	lh	0.093	-0.019	0.014	0.02	**0.001**	**0**
rostralanteriorcingulate	rh	0.015	-0.032	0.000	0.00	0.561	0.58
rostralmiddlefrontal	lh	0.051	-0.015	0.000	0.00	**0.001**	**0**
rostralmiddlefrontal	rh	0.043	-0.008	0.112	0.15	**0.019**	**0.04**
superiorfrontal	lh	0.032	-0.030	0.000	0.00	**0.047**	0.07
superiorfrontal	rh	0.030	-0.050	0.000	0.00	0.114	0.14
superiorparietal	lh	0.053	-0.017	0.000	0.00	**0**	**0**

6 Gradunwarp gradient distortion corrected data

6.1 Vertex-wise analysis of gradunwarp distortion-corrected ICC

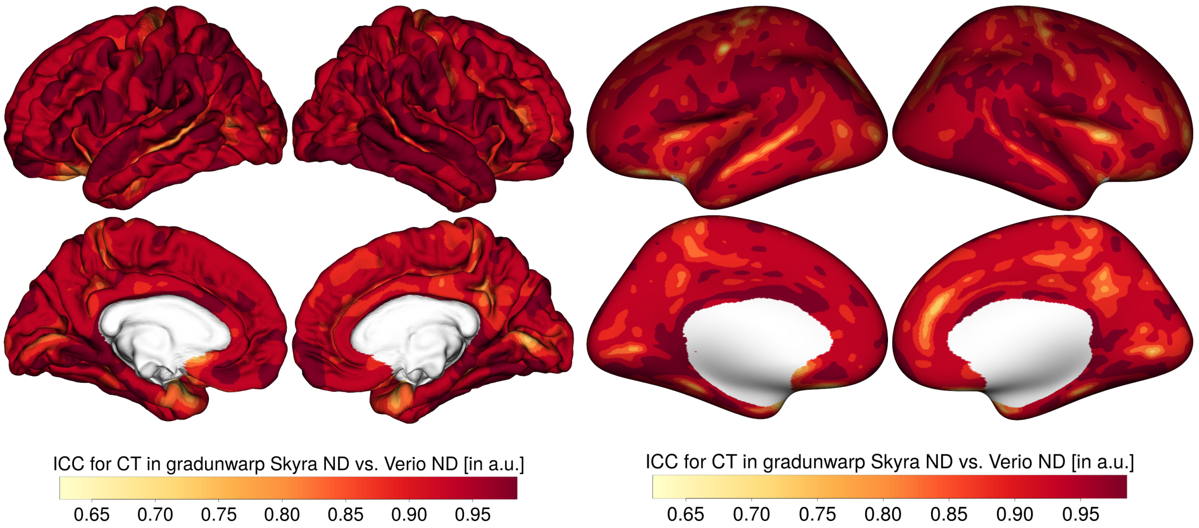

Figure 8: ICC for cortical thickness (CT) of gradunwarp Skyra ND and Verio ND on white surface (left panel) and inflated surface (right panel)

6.2 Vertex-wise analysis of gradunwarp distortion-corrected PD

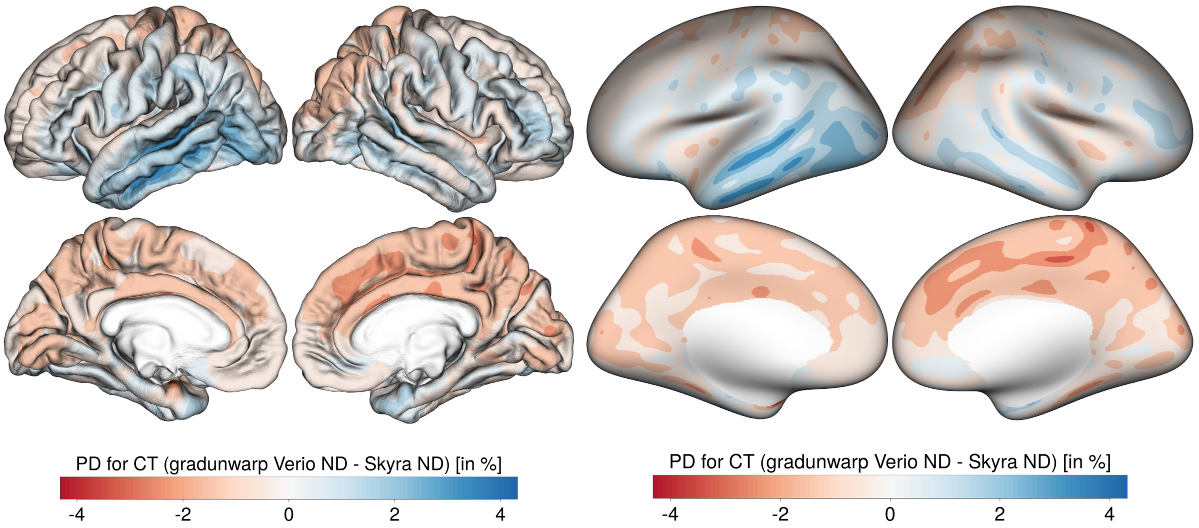

Figure 9: PD for cortical thickness (CT) of gradunwarp Skyra ND and Verio ND on white surface (left panel) and inflated surface (right panel) Positive values: Verio>Skyra , negative values: Verio<Skyra))

7 Skyra D versus Verio ND analysis

8 QA differences

When comparing the acquisitions with and without vendor-provided online gradient distortion correction on the Skyra scanner (D and ND), we observed that the distortion correction increased CNR (β=-0.113, p <

Table 5: Reliability and percent difference for CT from gradunwarp distortion corrected data (T<0 reflects Skyra >Verio , T>0 reflects Verio >Skyra)

ROI	hemi	ICC	lower ICC	upper ICC	PD	T	p	adj.p
bankssts	lh	0.849	0.849	0.873	1.90	9.56	0.00	**0**
bankssts	rh	0.939	0.909	0.947	0.87	5.71	0.00	**0**
caudalanteriorcingulate	lh	0.966	0.957	0.971	-1.06	-6.32	0.00	**0**
caudalanteriorcingulate	rh	0.931	0.909	0.935	-1.72	-10.45	0.00	**0**
caudalmiddlefrontal	lh	0.914	0.873	0.933	-0.35	-2.30	0.02	**0.03**
caudalmiddlefrontal	rh	0.935	0.909	0.957	0.01	0.03	0.97	0.97
cuneus	lh	0.911	0.884	0.940	-0.91	-3.97	0.00	**0**
cuneus	rh	0.935	0.926	0.948	-0.98	-5.04	0.00	**0**
entorhinal	lh	0.903	0.902	0.927	-0.21	-0.57	0.57	0.64
entorhinal	rh	0.957	0.945	0.971	-0.15	-0.67	0.50	0.57
fusiform	lh	0.908	0.905	0.927	0.25	1.65	0.10	0.16
fusiform	rh	0.941	0.932	0.956	-0.35	-2.91	0.00	**0**
inferiorparietal	lh	0.886	0.845	0.888	0.31	1.91	0.06	0.1
inferiorparietal	rh	0.903	0.891	0.926	-0.89	-6.30	0.00	**0**
inferiortemporal	lh	0.889	0.875	0.910	1.47	10.57	0.00	**0**
inferiortemporal	rh	0.947	0.932	0.965	0.16	1.29	0.20	0.27
isthmuscingulate	lh	0.975	0.972	0.981	-0.80	-5.27	0.00	**0**
isthmuscingulate	rh	0.951	0.949	0.957	-1.15	-7.09	0.00	**0**
lateraloccipital	lh	0.884	0.863	0.913	1.01	5.07	0.00	**0**
lateraloccipital	rh	0.924	0.908	0.935	-0.20	-1.16	0.25	0.31
lateralorbitofrontal	lh	0.875	0.829	0.902	0.49	2.82	0.01	**0.02**
lateralorbitofrontal	rh	0.888	0.882	0.914	0.27	1.17	0.25	0.31
lingual	lh	0.937	0.917	0.948	-0.86	-5.21	0.00	**0**
lingual	rh	0.926	0.884	0.939	-0.74	-4.08	0.00	**0**
medialorbitofrontal	lh	0.796	0.769	0.810	0.35	1.02	0.31	0.38
medialorbitofrontal	rh	0.891	0.860	0.908	0.19	0.87	0.38	0.45
middletemporal	lh	0.876	0.806	0.909	1.75	12.31	0.00	**0**
middletemporal	rh	0.948	0.944	0.954	0.37	2.94	0.00	**0**
parahippocampal	lh	0.974	0.970	0.978	0.24	1.51	0.13	0.2
parahippocampal	rh	0.964	0.951	0.966	-0.30	-1.76	0.08	0.13
paracentral	lh	0.893	0.859	0.903	-1.33	-8.10	0.00	**0**
paracentral	rh	0.853	0.806	0.882	-1.84	-11.69	0.00	**0**
parsopercularis	lh	0.947	0.938	0.962	-0.01	-0.07	0.94	0.95
parsopercularis	rh	0.944	0.946	0.963	0.02	0.13	0.90	0.94
parsorbitalis	lh	0.926	0.919	0.944	-0.14	-0.76	0.45	0.52
parsorbitalis	rh	0.931	0.878	0.945	0.03	0.13	0.89	0.94
parstriangularis	lh	0.911	0.885	0.932	0.01	0.10	0.92	0.95
parstriangularis	rh	0.913	0.860	0.915	0.06	0.34	0.74	0.8
pericalcarine	lh	0.884	0.871	0.905	-1.11	-3.64	0.00	**0**
pericalcarine	rh	0.869	0.845	0.886	-0.84	-2.23	0.03	0.05
postcentral	lh	0.950	0.942	0.962	-0.13	-0.88	0.38	0.45
postcentral	rh	0.949	0.934	0.966	-0.47	-3.32	0.00	**0**
posteriorcingulate	lh	0.950	0.942	0.961	-1.31	-10.36	0.00	**0**
posteriorcingulate	rh	0.900	0.883	0.924	-1.76	-14.37	0.00	**0**
precentral	lh	0.919	0.898	0.922	-0.40	-2.89	0.00	**0**
precentral	rh	0.934	0.889	0.940	-0.18	-1.49	0.14	0.21
precuneus	lh	0.853	0.817	0.854	-1.20	-7.32	0.00	**0**
precuneus	rh	0.832	0.798	0.842	-1.58	-10.59	0.00	**0**
rostralanteriorcingulate	lh	0.892	0.841	0.881	-0.23	-0.95	0.35	0.42
rostralanteriorcingulate	rh	0.905	0.882	0.916	-0.67	-3.25	0.00	**0**
rostralmiddlefrontal	lh	0.893	0.868	0.913	-0.36	-2.32	0.02	**0.03**
rostralmiddlefrontal	rh	0.853	0.830	0.891	0.25	1.46	0.15	0.22
superiorfrontal	lh	0.905	0.897	0.923	-0.86	-6.65	0.00	**0**
superiorfrontal	rh	0.876	0.850	0.889	-1.13	-8.47	0.00	**0**

Table 6: Reliability and percent difference for cortical thickness from Skyra D versus Verio ND data (T<0 reflects Skyra >Verio , T>0 reflects Verio >Skyra)

ROI	hemi	ICC	lower ICC	upper ICC	PD	T	p	adj.p
bankssts	lh	0.841	0.807	0.889	1.97	9.88	0.00	**0**
bankssts	rh	0.909	0.858	0.937	1.47	8.92	0.00	**0**
caudalanteriorcingulate	lh	0.958	0.944	0.965	-1.20	-6.60	0.00	**0**
caudalanteriorcingulate	rh	0.880	0.834	0.898	-2.45	-11.47	0.00	**0**
caudalmiddlefrontal	lh	0.884	0.870	0.921	-0.91	-5.31	0.00	**0**
caudalmiddlefrontal	rh	0.895	0.868	0.913	-0.37	-2.16	0.03	**0.04**
cuneus	lh	0.884	0.885	0.922	1.25	4.85	0.00	**0**
cuneus	rh	0.919	0.918	0.928	0.42	1.70	0.09	0.12
entorhinal	lh	0.916	0.905	0.940	-0.15	-0.51	0.61	0.67
entorhinal	rh	0.937	0.919	0.953	-0.19	-0.69	0.49	0.55
fusiform	lh	0.883	0.849	0.911	0.96	6.00	0.00	**0**
fusiform	rh	0.929	0.911	0.937	0.42	3.37	0.00	**0**
inferiorparietal	lh	0.863	0.794	0.903	1.21	7.78	0.00	**0**
inferiorparietal	rh	0.930	0.902	0.933	0.39	2.88	0.00	**0**
inferiortemporal	lh	0.858	0.800	0.853	1.74	10.85	0.00	**0**
inferiortemporal	rh	0.910	0.878	0.921	1.07	8.40	0.00	**0**
isthmuscingulate	lh	0.979	0.974	0.981	-0.39	-2.50	0.01	**0.01**
isthmuscingulate	rh	0.950	0.937	0.960	-0.96	-5.47	0.00	**0**
lateraloccipital	lh	0.742	0.689	0.765	3.23	15.52	0.00	**0**
lateraloccipital	rh	0.857	0.825	0.908	1.86	9.34	0.00	**0**
lateralorbitofrontal	lh	0.860	0.842	0.901	0.73	4.24	0.00	**0**
lateralorbitofrontal	rh	0.794	0.789	0.842	0.65	2.42	0.02	**0.03**
lingual	lh	0.918	0.905	0.935	0.78	3.99	0.00	**0**
lingual	rh	0.923	0.905	0.933	0.61	3.21	0.00	**0**
medialorbitofrontal	lh	0.849	0.822	0.902	1.07	4.12	0.00	**0**
medialorbitofrontal	rh	0.870	0.790	0.906	-0.08	-0.37	0.71	0.74
middletemporal	lh	0.875	0.861	0.896	1.76	11.94	0.00	**0**
middletemporal	rh	0.905	0.888	0.923	1.26	9.41	0.00	**0**
parahippocampal	lh	0.964	0.954	0.973	0.73	3.87	0.00	**0**
parahippocampal	rh	0.962	0.951	0.967	0.02	-0.02	0.99	1
paracentral	lh	0.824	0.732	0.831	-2.34	-12.51	0.00	**0**
paracentral	rh	0.710	0.677	0.761	-3.16	-13.62	0.00	**0**
parsopercularis	lh	0.949	0.941	0.959	-0.16	-1.30	0.20	0.25
parsopercularis	rh	0.935	0.926	0.953	0.07	0.45	0.65	0.7
parsorbitalis	lh	0.930	0.908	0.939	0.29	1.62	0.11	0.14
parsorbitalis	rh	0.942	0.935	0.963	0.20	0.95	0.34	0.41
parstriangularis	lh	0.913	0.889	0.941	0.28	1.90	0.06	0.08
parstriangularis	rh	0.901	0.874	0.931	0.36	2.02	0.05	0.07
pericalcarine	lh	0.838	0.829	0.862	1.65	4.77	0.00	**0**
pericalcarine	rh	0.796	0.716	0.826	2.42	5.93	0.00	**0**
postcentral	lh	0.925	0.907	0.937	0.94	5.78	0.00	**0**
postcentral	rh	0.929	0.900	0.949	0.98	6.50	0.00	**0**
posteriorcingulate	lh	0.924	0.907	0.927	-1.55	-9.58	0.00	**0**
posteriorcingulate	rh	0.819	0.781	0.844	-2.43	-13.68	0.00	**0**
precentral	lh	0.893	0.880	0.899	-1.03	-7.25	0.00	**0**
precentral	rh	0.933	0.909	0.933	-0.73	-4.85	0.00	**0**
precuneus	lh	0.910	0.845	0.914	0.00	-0.04	0.97	1
precuneus	rh	0.884	0.860	0.913	-0.86	-5.63	0.00	**0**
rostralanteriorcingulate	lh	0.814	0.792	0.866	-0.42	-1.36	0.18	0.23
rostralanteriorcingulate	rh	0.920	0.906	0.931	-0.80	-4.24	0.00	**0**
rostralmiddlefrontal	lh	0.892	0.868	0.915	-0.53	-3.41	0.00	**0**
rostralmiddlefrontal	rh	0.857	0.826	0.894	0.00	0.00	1.00	1
superiorfrontal	lh	0.805	0.799	0.835	-2.01	-13.08	0.00	**0**
superiorfrontal	rh	0.687	0.650	0.702	-2.67	-16.08	0.00	**0**

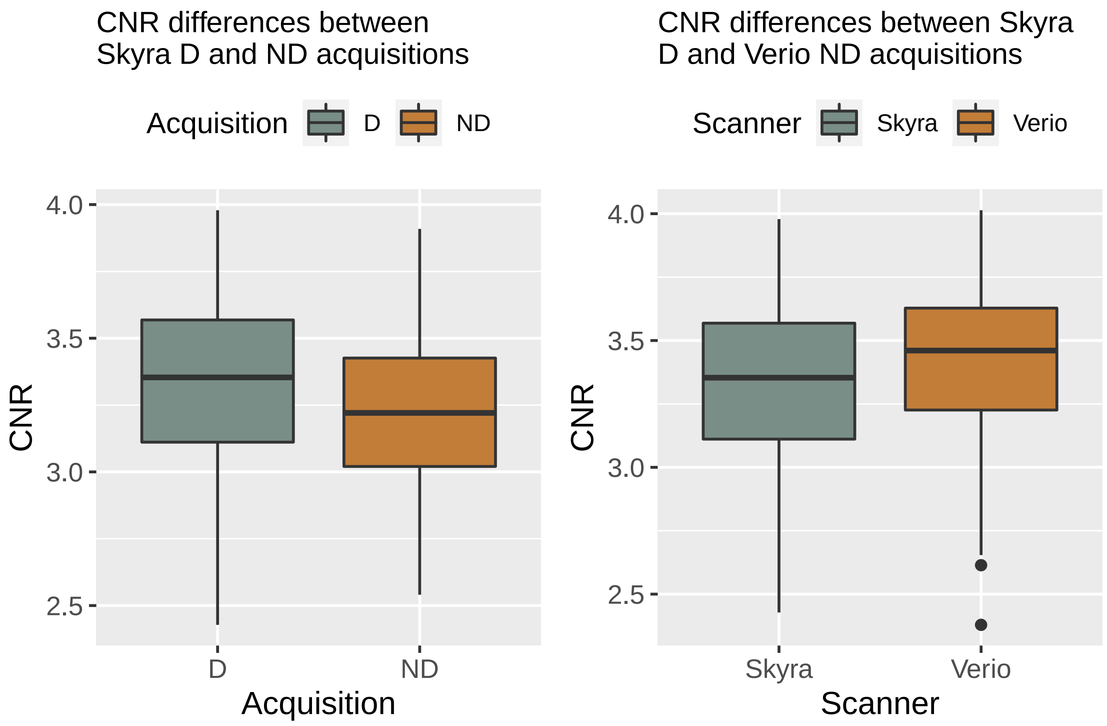

Figure 10: CNR differences between the Skyra D and ND acquisitions (left panel) and between Skyra D and Verio ND acquisitions (right panel), showing higher CNR irrespective of gradient distortion on the Verio scanner

0.001, see Figure 10, left panel).

When comparing Verio ND and Skyra D, we also see higher CNR on the Verio scanner (β=0.088, $p < 0.001$, see Figure 10, right panel), which is expected given the CNR difference between Verio ND and Skyra ND reported in the main manuscript.
