# Supplementary material for: Estimating the effect of a scanner upgrade on measures of grey matter structure for longitudinal designs
Source: PLoS One. 2021 Oct 5;16(10):e0239021. doi: 10.1371/journal.pone.0239021 (PMC8491918; doi:10.1371/journal.pone.0239021)
Supplement: Attachment — Submitted filename: Letter_to_reviewers_FINAL.docx [file pone.0239021.s002.docx]

Answers to Reviewers

*We thank both reviewers for their thorough review of our work and the very helpful recommendations. Please see below for our replies, printed in italics. Changes to the manuscript are highlighted in yellow. We changed the manuscript according to the reviewer’s suggestions and provide a version, in which all track changes are highlighted in yellow.*

**Reviewer 1:**

**1. System differences**
**Please add a description of what was the difference in the systems, e.g. were the same coil used for receive? What are the difference in transmit amplitud and changes, receive were the same coil used, number of channels.
The lac of information of what differs between the systems makes it difficult to evaluate what could cause the differences in gray matter volumes.**

*We agree that more information on the systems would make it easier for the reader to understand the differences we describe. We thus added a table which shows scanner and image acquisition parameters to the Methods section of the manuscript (see Table 1 below). Overall, the scanners differed in various technical parameters: software version, transmit coil and transmit-receive electronics, which is reflected in the transmission amplifier reference amplitude. Gradients and field strength were identically constructed, but might still slightly vary between scanners. Similarly, we used two 32-channel receive coils which differed in plug system and could thus not be interchanged. Sequence parameters were the same on both scanners, yet again, we do not have access to possible changes in reconstruction algorithms between software versions.*

Table 1, added to the manuscript

| MRI scanner | Verio | Skyra |
| --- | --- | --- |
| Nominal field strength | 3 Tesla | 3 Tesla |
| System software version | syngo MR B17 | syngo MR E11 |
| Tx/Rx coil | Body coil: Tim Tx Trueform [102x8], [102x18], [102x32]  32-channel head coil | Bodycoil:Tim 4G [204x48], [204x64]  32-channel head coil |
| Transmission amplifier reference amplitude (mean/standard deviation in V) | 432/27.8 | 311/15.7 |
| Gradient system | VQ Gradients 45 mT/m @ 200 T/m/s | VQ Gradients 45 mT/m @ 200 T/m/s |
| Coil combination algorithm | adaptive coil combination | adaptive coil combination |
| Imaging sequence | Vendor-implementation of MPRAGE with standardized parameters according to ADNI-3 | Vendor-implementation of MPRAGE with standardized parameters according to ADNI-3 |
| Parallel imaging method | Grappa with iPAT: 2 | Grappa with iPAT: 2 |
| TE (ms) | 2.98 | 2.98 |
| Flip angle | 9 | 9 |
| Acquisition time (min:sec) | 5:10 | 5:10 |

**2. Comparing volumes**
**I have doubts about using ICC for comparing the estimated gray matter volumes the estimated ICC values seems to be strongly dependent on the range of the subjects volumes. I.e. the higher ICC for caudate compared to the ICC for amygdala could be that the subjects have a higher variation in caudate volume than the variation in amygdala volume. As I see it Bland Altman analyses would be more suited for comparing volumes estimate using the different systems. Pleas add and discuss Bland Altman plots for the gray matter volumes.**

*We agree with the reviewer that Bland Altman analyses are a suitable addition to our ICC analysis. We used ICC because it is a standard measure to assess agreement between raters, which has been used in other studies comparing GMV (test-retest) reliability [Plitman et al. 2020 and Potvin et al., 2019]. Yet, we are aware that ICC depends on the amount of between-person variance and may not be comparable when measures (e.g. amygdala and caudate volume) differ in variability across participants. Thus, we added exemplary Bland-Altman plots for superior frontal and lateral occipital cortical thickness (see Figures below), as well as amygdala and caudate to the main manuscript and a plot showing mean and difference in all subcortical volumes to the supplementary Material. In the results section for the cortical analysis on p. 16, it now reads: “Bland-Altman plots confirmed the bias of Verio versus Skyra measurements. Exemplary plots of superior frontal and lateral occipital regions are shown in Figure 6. For the superior frontal region, CT, CA and CV are larger for Skyra compared to Verio with 95% of CT differences were between -0.04 and -0.12 mm, 95% of CA differences were between -103.12 and -285.25* $mm^{2}$*, and 95% of CV differences were between -694.23 and -1841.61* $mm^{3}$*. The inverse pattern was present in the lateral occipital region with 95% of CT differences were between 0.03 and -0.07* $mm$*, 95% of CA differences were between -55.32 and -156.55* $mm^{2}$*, and 95% of CV differences were between -86.04 and -772.28* $mm^{3}$*”*

*On p. 17, we further added “Bland-Altman plots for subcortical regions confirmed the systematic bias and further indicated that differences in variability between subjects influenced ICC estimates. For example, there was high between-subject variability in the Thalamus, so that, despite large differences between measurements, ICC was high. Similarly, differences between scanners were less pronounced in the Accumbens, yet, due to lower between-subject variability the ICC of this region was lower (see Figure 7, and Figure 1 in the Supplementary Material). For the Accumbens, 95% of differences between scanners were between -143.02 and 37.38* $mm^{3}$*, for Thalamus 95% of differences were between -914.4 and 233.85* $mm^{3}$*.”*

*In the discussion on p.24, we now discuss the limitations of the ICC: “ICC is a common yet somewhat flawed measure of reliability. ICC does not reflect differences in inter-individual variability, as underlined by Bland-Altman plots of subcortical volumes, and was high in this study even though substantial bias was present.”*


**3. QA measures**
**It is interesting that the Verio scanner seems to perform better for all images quality than the Skyra fit scanner. However, I lac a discussion of what the underlying reason for this. It is also related to the lac of information of the systems. E.g. could this be due to differences that the MPRAGE sequence are optimised for the Verio system eg flip angle optimised for the transmit system of the Verio, grappa factor optimised for the verio head coil etc.**

*We agree with the reviewer and extended our analysis to signal-to-noise ratio which is also provided by mriqc. Here, we saw no significant SNR difference between Verio and Skyra. Yet, we found higher grey-to-white matter contrast as indicated by the higher CNR and CJV in Verio compared to Skyra.
The MPRAGE sequence from the ADNI-3 protocol is recommended for both Verio and Skyra (*[*http://adni.loni.usc.edu/methods/documents/mri-protocols/*](http://adni.loni.usc.edu/methods/documents/mri-protocols/)*) and was performed with identical settings in this study. Thus, we do not believe that the quality differences relate to specific settings in the sequence such as flip angle or acceleration factor. Instead, the small, yet significant differences in gray-white matter contrast likely arise from variation in transmit and receive hardware as well as software. We added Table 1 to describe differences between scanners in more detail on p. 8. The analysis of SNR is described in the Methods section on p.11: “We used signal-to-noise ratio (SNR) to assess overall signal quality and compared contrast-to-noise ratio (CNR) to quantify the difference between grey and white matter intensity distributions. Furthermore, we used coefficient of joint variation (CJV) which also reflects grey-to-white matter contrasts and entropy focus criterion (EFC) to describe the amount of ghosting and blurring induced by head motion [24].” and in the Results section on p. 18: “We found that there was no significant difference in SNR between Verio and Skyra (*$\beta$*=0.06, p = 0.07).”*

**4. Figure 6**
**I do not get the CNR data to match. In the left panel Skyra ND data goes between 3 to 3.4 and in the right panel the Skyra ND CNR values goes between 3.1 and 3.6.**

*You are right, thank you for pointing this out, there was an error in the generation of the plots. According to the general change in the structure of the manuscript (see Answer to Reviewer 2), we now present Skyra ND versus Verio ND as the main analysis, and moved the comparison of Skyra D and Verio ND to the supplementary Material (p.14). Now, Skyra D CNR values are the same in both panels.*

**Minor**
**5.** **Please present Age in Median and range, mean and sd are more suitable parameters that are normal distributed. The same goes for time between scanning.**

*We updated the presentation of age and time between scanning in the Methods section, please see p. 6, where it now reads: “121 healthy participants (median age = 28 years, range = 19 - 54 years [...]” and “The median time between sessions was 1.92 months (range: 0.12 - 4.56 months)”*

**6.** Page 11 section Comparison to previous upgrade studies, In this section the gray matter volume are abbreviated some time as GM volume some times as GV some times as GMV please be consistent.

*Thank you for spotting this issue. We modified the manuscript to be more consistent.*

Reviewer #2
**Major comments**

**1.** **The authors are analyzing the data here in two ways (cf. Fig 1.) by comparing Verio ND to Syra D data and by comparing Verio ND with gradunwarp to Skyra ND with gradunwarp. This is introduced on page 4, line 99-102. However, these lines are in the subsection on details regarding the performed MRI acquisitions.
a. I believe it makes much more sense that this is included in the Analysis section which allows then to list all the different analyses performed using the described metrics (ICC, PD, etc…).
b. The first comparison, referred to as “main analysis” in the manuscript (Imaging sequence subsection) is however done on data from the Skyra with gradient distortions correction and from the Verio without gradient distortion correction. As pointed out by the authors themselves, cf. subsection “Gradient distortion correction”, gradient distortion correction has an impact on the final image, as described in referenced literature, so I am missing the reason to do this comparison here.
I would have found more logical to compare the data sets Skyra ND with Verio ND, because this makes a direct comparison between data which have seen supposedly the same steps during image reconstruction and make a nice introduction/motivation to the analysis described in subsection “Effect of offline gradient distortion correction”
I am therefore asking the authors to add this comparison and to motivate the reason to do a comparison between the data sets Skyra D with Verio ND.**

*We agree with the points the reviewer raised. We moved the description of the performed comparisons to the section “Analysis”, and changed the main analysis to be the comparison between Verio ND and Skyra ND. We also believe that it makes more sense to compare images at a similar stage of image reconstruction, especially knowing that gradient distortions can have a large impact on image quality. On p. 19, this is illustrated by the comparison of ICC between Verio ND vs Skyra ND, gradunwarp Verio ND vs. Skyra ND and the “default” analysis Verio ND vs Skyra D. “In addition, we saw that the systematic bias was largest when comparing the default scanner output images Skyra D (online gradient distortion correction) and Verio ND (mean ICC gradunwarp Skyra ND vs Verio ND: 0.91, mean ICC Skyra ND vs Verio ND: 0.91, mean ICC Skyra D vs Verio ND: 0.89, ANOVA F-test: F = 3.7, p = 0.026).” Here, Verio ND vs. Skyra D performed slightly, yet significantly worse.*

Please find the modified Figure 1 on p. 11 and below, and all changes in the updated manuscript highlighted in yellow.

**2. Was the same coil used on both scanners? Please comment, discuss the possible implications with your findings. I speculate this could have a strong influence on the differences observed regionally and why you do not have the same findings as in the other studies referenced in the discussion, [33] and [34].**

*Yes, a 32-channel head coil was used on both scanners. We could not use the same head coil on both, because the scanners differed in plug system. We thus added the possibility that head coils influenced our results to the Discussion on p. 23, where it now reads: “Taken together, we believe the systematic biases between Verio and Skyra stem from both scaling and image intensity differences, and are related to both differences in scanner hardware, e.g. receiver head coils, and software, e.g. differences in reconstruction algorithms.”*

**3.** **Was the same coil combination algorithm used (sum of squares or adaptive coil combination) which is available on both scanners?**
**I am aware that the coil combination algorithm has evolved from VB to VE which should not impact so much MPRAGE images in the brain, but is an example that not only hardware changes can have an effect but also pure software updates. In the discussion section, line 380, you are limiting yourself there to hardware. Purely software upgrades can also have impacts on the final obtained image from the scanner because of changes in the sequence or the image reconstruction.**

*We added Table 1 to the manuscript to give the reader an overview of differences between scanners and acquisitions (see also reply to comment 1 of reviewer #1). The same coil combination algorithm (adaptive coil combination) was used for both. We however agree that software upgrades are important and extended the discussion accordingly. It now reads, for example on p. 23:”Taken together, we believe the systematic biases between Verio and Skyra stem from both scaling and image intensity differences, and are related to both differences in scanner hardware, e.g. receiver head coils, and software, e.g. differences in reconstruction algorithms.”*

**4. As stated, it could have been adequate to add a test re-test on a subset of the participants on the two scanners to help to put perspective on the given numbers for the scan-rescan reliability. If such a data subset exists, it would really roundup this study nicely to address repeatability. Are there published articles which have investigated the repeatability of the MPRAGE on one of both scanners used in this study and where a similar analysis is performed on grey matter structure?**

*We agree with the reviewer that a retest-acquisition of the same participants on the same scanner would have been ideal to round up this study and assess test-retest reliability. Unfortunately, we did not have the opportunity to acquire this data due to time and personnel constraints, i.e. that the follow-up of the cohort study was scheduled already. Yet, previous studies have reported percent difference values for cortical thickness from 1-5% and for subcortical volumes from 2 – 4 % (but no bias) for test-retest acquisitions on Skyra and Verio (Jovivich et al., 2013 and Yan et al., 2020).*

*We have expanded the Limitations section in the manuscript (p. 24) accordingly, where it now reads: “Another limitation is that we did not randomize the order of participants across scanners and that we could not assess test-retest reliability as we only performed one scan on each system. Yet, previous studies indicated that PD of cortical and subcortical GM are comparable to our results (i.e. PD for subcortical volumes around 2-4 % on Skyra and Verio scanners.“*

**Minor comments**

We adressed all minor comments by the reviewer throughout the manuscript.
1. page 3 line 55: Siemens Healthcare MAGNETOM Verio to Skyrafit. Please consisting of the naming of the scanners throughout the manuscript.

*We changed to Verio and Skyra whenever referring to the scanners. We give the exact product names in the Methods section and added “Scanners are referred to as Verio and Skyra throughout the manuscript.”.*
2. page 3 line 64: 3 T MAGNETOM Verio vs Skyrafit
3. page 3 line 67: Tim 4G, new bdy coil
4. page 3 line 66-67: the information in parentheses is not needed here or as an alternative the reference the product brochure which lists the hardware change <https://cdn0.scrvt.com/39b415fb07de4d9656c7b516d8e2d907/1800000000279602/0c4ab340a772/MRI-MAGNETOM-Verio-Upgradebrochure_1800000000279602.pdf>

*We included more details on hardware and software changes in Table 1 as recommended by both reviewers, and referenced the brochure stored in our github repository https://github.com/fBeyer89/life_upgrade.*
5. page 3 line 68: on the upgrade of the Verio to a Skyrafit ([...]
6. page 3 line 68 to 70: Sentence is confusing here. Could be removed or integrated to the Discussio*n. The sentence has been removed.*
7. page 4, line 80-81: the software versions VB and VE are not subsequent with each other. *We removed this part of the sentence.*
8. page 4, line 81: Siemens Healthcare, Erlangen, Germany
9. page 4, line 95: remove reference to a paper not yet submitted, available
Repalce with e.g. " [...] analysis results will be presented in subsequent publication(s)."
10. page 4, line 110:"correction" should be removed, should'nt it? *No, it’s the correction of gradient distortions.*
11. line 120: add a reference to M.F. Glasser et al. / NeuroImage [80 (2013) 105](callto:80 (2013) 105)–124 for the graduwarp code and J. Jovicich et al. / NeuroImage [30 (2006) 436](callto:30 (2006) 436)–443 as a reference for the method itself*. We added the references to the manuscript.*
12. line 130: remove replace ',' by 'and'
13. line 134: remove ','
14. line 146: in parenthenses (version 0.15 ...)
15. line 153: Shift "As outcomes" to the end of the sentence.
16. I found this paper
N.K. Dinsdale, M. Jenkinson and A.I.L. Namburete, NeuroImage 228 (2021)
which seems as well promising and should be cited alongside the ComBat method referenced in the discussion to tackle the problem described in your study. *We agree, and included the paper in the Discussion on p. 16.*
17. It would be helpful that the abbreviations defined in the manuscript are written out again in the figure captions to help the reader to understand what e.g. ICC or PD stands for. *We adapted the figure captions accordingly.*
