# Supplementary material for: Estimating the effect of a scanner upgrade on measures of grey matter structure for longitudinal designs
Source: PLoS One. 2021 Oct 5;16(10):e0239021. doi: 10.1371/journal.pone.0239021 (PMC8491918; doi:10.1371/journal.pone.0239021)
Supplement: Attachment — Submitted filename: Letter_to_Reviewers_19.07.21.docx [file pone.0239021.s003.docx]

Answers to Reviewers

*We thank the reviewers for their positive evaluation and the additional comments to the revised version. We changed the manuscript according to the reviewer’s suggestions and provide a version, in which all track changes are highlighted in yellow.*

**Reviewer #1:** The authors have addressed all my concerns, and this version is more readable and better structured. I only have minor comments regarding the Bland Altman plots in figure 6 and 7.

There are a bit much text in the figure that make it hard to read. E.g. The region name could be moved from being repeated 3 times in figure 6 the title of the x-axis to be written one time above each column. Likewise the title of the y-axis could be omitted for the second column (both figure 6 and 7).

Moreover, the figure would bee more readable if the scale of the y-axis were the same for all regions and the bias would be more readable if the scales were centred around 0 figure (both figure 6 and 7).

In figure 6 the y-label is cooped and it is started that the distance is in m not mm.

*We thank the reviewer for the suggestions which indeed made the figures more easily readable. We abstained from making the y-axis the same for all regions, because the scales of CT, CA and CV differ largely. Yet, we used the same y-axis limits for both cortical regions, and all subcortical regions. Please see the updated figures in the manuscript.*

**Reviewer #2:** Dear author, thank you for addressing the reviewers’ comments. I have only some minor comments on the revised manuscript which you will find below. Thank you for the interesting manuscript.

Minor comments: Table 1, System software version : Please add, if possible, the complete software version name, e.g. syngo MR B17A or E11A. For example, there is a big difference between the versions E11C and E11A. Please update correspondingly line 81 and 82. Given the dates, I believe that it must be B17A and E11A.

*Thank you for pointing out the importance of the exact version specification. We updated the version names to syngo MR B17A and E11C throughout the manuscript.*

Table 1, Tx/Rx coil line: Please state the correct number of RF channel for your systems. You are listing the various RF configuration possibilities. It is for the Verio either [102x8], [102x18] or [102x32]. Because you are using the 32 channel head coil it can only the [102x32] RF configuration. Same for the Skyra, it can only be one of the two listed RF configuration.

*We apologize for this mistake. We changed the table to include the correct RF configurations [102 x 32] for Verio and [204x64] for Skyra.*

Line 55: from MAGNETOM Verio to MAGNETOM Skya fit.

Line 81 and 82: Magnetom in capital letters.

Line 81 and 82: please change to “ […] MAGNETOM Verio with software version syngo MR B17 ([…] “ and “ […] MAGNETOM Verio with software version syngo MR E11 ([…] “. Please add the correct software version as in Table 1.

Line 85 to 86: would weeks not be a better unit instead of months?
*We changed the manuscript according to the reviewer’s suggestions.*

Line 96: “Further, the scanner were not issue of an actual […]” Could it be that part of the sentence is missing?
*We clarified this sentence by changing it to* “In this study, the Verio scanner did not undergo an actual upgrade and the scanners therefore differed in the main B0-field and other hardware components.”

Line 134: Here you write gradunwrap not in italic but in line 140 you write it in italic. In order to be consistent throughout the manuscript, I suggest introducing the names of all used software package in italic, e.g. FreeSurfer, psy, etc… Line 276: Two points after each other, only one is needed to finish the sentence.
*We agree with the reviewer and changed all software tools and packages to be shown in italics.*

Line 401: no comma after “one study”. Please cite the paper of this study separately for clarity.
*We removed the comma and cited the studies separately as suggested.*
